# Supplementary material for: Mean Corpuscular Volume Is Correlated with Liver Fibrosis Defined by Noninvasive Blood Biochemical Indices in Individuals with Metabolic Disorders Aged 60 Years or Older
Source: J Clin Med. 2025 Jul 2;14(13):4680. doi: 10.3390/jcm14134680 (PMC12251042; doi:10.3390/jcm14134680)
Supplement: Supplementary file 1 [file jcm-14-04680-s001.zip › jcm-3692424-supplementary.pdf]

Table S1. Multiple regression analysis including identified variables and medications used for determinants of MASLD indices in overall subjects

	HSI			FIB-4 index			APRI			NFS		
Variables	VIF	*t* value	*p* value	VIF	*t* value	*p* value	VIF	*t* value	*p* value	VIF	*t* value	*p* value
Age	1.643	-9.40	<0.001	1.445	12.53	<0.001	-	-	-	1.715	18.41	<0.001
Male	1.367	-6.01	<0.001	-	-	-	-	-	-	-	-	-
BMI	1.393	54.96	<0.001	-	-	-	1.213	2.81	0.005	1.444	12.10	<0.001
LDL-C	-	-	-	1.228	-3.19	0.002	-	-	-	1.293	-2.80	0.005
HDL-C	-	-	-	-	-	-	1.175	2.26	0.024	1.253	2.82	0.005
HbA1c	1.463	3.93	<0.001	-	-	-	-	-	-	1.542	-2.47	0.014
Cr	1.278	-2.14	0.033	-	-	-	-	-	-	1.163	2.86	0.004
Hypertension	-	-	-	1.840	-0.60	0.548	-	-	-	-	-	-
Diabetes	2.040	7.84	<0.001	-	-	-	-	-	-	2.076	11.84	<0.001
ALB	1.377	4.11	<0.001	1.243	-1.99	0.047	-	-	-	1.281	-10.42	<0.001
Hct	1.618	1.55	0.122	-	-	-	-	-	-	-	-	-
MCV	-	-	-	1.289	4.61	<0.001	1.193	4.30	<0.001	1.367	3.06	0.002
ARB or ACEi	1.279	-0.76	0.447	1.554	-0.45	0.650	1.245	-2.59	0.010	1.274	0.01	0.988
CCB	1.249	0.13	0.901	1.445	-0.77	0.442	1.227	0.38	0.705	1.252	-1.70	0.090
β blocker	1.153	1.41	0.159	1.155	0.60	0.550	1.139	0.82	0.412	1.162	1.48	0.139
MR antagonist	1.111	1.05	0.292	1.093	0.40	0.692	1.080	-0.61	0.542	1.101	-1.52	0.129
Statin	1.131	1.43	0.155	1.189	-1.27	0.204	1.089	-0.07	0.948	1.204	-1.16	0.247
Ezetimibe	1.049	1.58	0.115	1.052	-1.32	0.188	1.048	0.20	0.845	1.053	-2.40	0.016
Antiplatelet	1.211	-1.25	0.212	1.157	0.92	0.356	1.152	1.42	0.156	1.180	2.08	0.038
SU or Glinide	1.282	-1.13	0.261	1.250	0.88	0.381	1.233	0.80	0.423	1.277	-0.26	0.793
Metformin	1.463	1.63	0.103	1.420	-3.34	<0.001	1.373	-2.93	0.003	1.468	-1.06	0.291
DPP-4i	1.991	-0.03	0.975	1.625	0.58	0.560	1.609	2.33	0.020	2.011	0.40	0.692

SGLT2i	1.405	1.12	0.263	1.256	-0.10	0.924	1.252	0.14	0.885	1.310	-0.36	0.722
α-GI	1.161	1.05	0.293	1.153	0.38	0.707	1.150	-0.03	0.973	1.158	0.58	0.561
Pioglitazone	1.071	-1.41	0.160	1.069	0.65	0.514	1.074	-0.06	0.949	1.076	1.63	0.104
Insulin	1.391	-1.24	0.215	1.148	-2.09	0.037	1.112	-0.44	0.660	1.396	-1.38	0.169
GLP-1RA	1.618	2.91	0.004	1.508	-0.11	0.915	1.510	1.89	0.059	1.628	-1.39	0.166

Table S2. Multiple regression analysis including identified variables and medications used for determinants of MASLD indices in subjects aged < 60 years

	HSI			FIB-4 index			APRI			NFS		
Variables	VIF	*t* value	*p* value	VIF	*t* value	*p* value	VIF	*t* value	*p* value	VIF	*t* value	*p* value
Age	1.317	-2.66	0.009	1.089	4.55	<0.001	-	-	-	1.326	5.48	<0.001
BMI	1.281	24.41	<0.001	-	-	-	-	-	-	1.361	6.97	<0.001
LDL-C	-	-	-	-	-	-	-	-	-	1.290	-2.61	0.010
Hypertension	-	-	-	1.806	-1.29	0.199	-	-	-	-	-	-
Diabetes	-	-	-	-	-	-	-	-	-	1.477	4.40	<0.001
ALB	1.140	2.54	0.012	-	-	-	-	-	-	1.175	-2.66	0.009
ARB or ACEi	1.694	0.96	0.340	1.889	-0.89	0.377	1.677	-0.93	0.353	1.696	-1.45	0.150
CCB	1.664	-0.71	0.481	1.777	0.56	0.577	1.590	0.27	0.787	1.713	0.08	0.938
β blocker	1.286	0.60	0.546	1.291	0.06	0.950	1.278	0.12	0.902	1.333	1.49	0.138
MR antagonist	1.133	0.42	0.674	1.132	-0.34	0.735	1.121	-0.24	0.812	1.148	-0.24	0.813
Statin	1.309	0.57	0.570	1.308	-0.62	0.538	1.279	0.12	0.907	1.332	-0.32	0.750
Ezetimibe	1.161	-0.09	0.928	1.165	0.17	0.863	1.154	0.14	0.892	1.164	-1.07	0.285
Antiplatelet	1.349	-0.75	0.452	1.328	-0.73	0.465	1.328	-1.34	0.181	1.356	0.65	0.514
SU or Glinide	1.189	-0.43	0.668	1.202	1.48	0.141	1.176	0.58	0.565	1.196	1.27	0.204
Metformin	1.346	1.50	0.135	1.343	-1.66	0.098	1.342	-1.01	0.316	1.413	-0.65	0.518
DPP-4i	1.435	-0.06	0.955	1.432	1.41	0.160	1.424	1.33	0.185	1.557	0.63	0.530
SGLT2i	1.342	1.02	0.308	1.332	-0.47	0.639	1.317	-0.29	0.774	1.385	-0.68	0.499
α-GI	1.264	0.34	0.731	1.259	-0.79	0.429	1.247	-0.84	0.405	1.266	0.94	0.350
Pioglitazone	1.102	-0.03	0.978	1.106	-0.60	0.550	1.097	-0.68	0.498	1.107	-0.47	0.637
Insulin	1.133	-0.21	0.830	1.135	-0.54	0.590	1.090	-0.18	0.859	1.162	-0.50	0.620
GLP-1RA	1.671	1.68	0.096	1.645	2.53	0.013	1.636	1.98	0.049	1.779	-0.22	0.830

Table S3. Multiple regression analysis including identified variables and medications used for determinants of MASLD indices in subjects aged ≥ 60 years

	HSI			FIB-4 index			APRI			NFS		
Variables	VIF	*t* value	*p* value	VIF	*t* value	*p* value	VIF	*t* value	*p* value	VIF	*t* value	*p* value
Age	1.371	-6.14	<0.001	1.233	10.26	<0.001	-	-	-	1.418	12.46	<0.001
Male	1.236	-6.81	<0.001	-	-	-	1.160	2.54	0.011	-	-	-
BMI	1.201	49.24	<0.001	-	-	-	-	-	-	1.260	9.14	<0.001
LDL-C	-	-	-	1.193	-3.37	<0.001	1.225	-2.13	0.034	1.265	-2.12	0.035
HDL-C	-	-	-	-	-	-	1.185	2.48	0.013	1.260	2.65	0.008
HbA1c	1.405	4.20	<0.001	-	-	-	-	-	-	1.466	-2.61	0.009
Cr	1.280	-2.36	0.019	-	-	-	-	-	-	1.185	3.02	0.003
Diabetes	2.200	8.04	<0.001	-	-	-	-	-	-	2.231	11.15	<0.001
ALB	1.295	3.81	<0.001	-	-	-	-	-	-	1.326	-10.42	<0.001
MCV	-	-	-	1.196	4.63	<0.001	1.180	4.29	<0.001	1.277	3.03	0.003
ARB or ACEi	1.229	-1.55	0.121	1.192	-0.15	0.884	1.194	-1.83	0.067	1.228	0.74	0.462
CCB	1.201	-0.08	0.933	1.185	-1.23	0.219	1.184	0.77	0.440	1.203	1.82	0.070
β blocker	1.185	-2.56	0.011	1.151	0.82	0.411	1.169	0.84	0.401	1.198	1.05	0.292
MR antagonist	1.132	0.96	0.338	1.106	0.58	0.559	1.106	-0.46	0.645	1.129	-1.29	0.199
Statin	1.118	0.97	0.334	1.156	-1.01	0.311	1.185	0.03	0.975	1.205	-0.62	0.533
Ezetimibe	1.043	1.56	0.120	1.045	-1.32	0.187	1.044	0.24	0.811	1.049	-2.08	0.038
Antiplatelet	1.214	-0.69	0.489	1.144	0.97	0.335	1.182	1.38	0.167	1.176	1.67	0.096
SU or Glinide	1.330	-0.64	0.520	1.291	0.18	0.854	1.272	0.43	0.669	1.321	-0.88	0.381
Metformin	1.514	0.98	0.325	1.459	-2.46	0.014	1.403	-2.55	0.011	1.523	-0.45	0.650
DPP-4i	2.090	0.21	0.837	1.678	-0.33	0.744	1.679	1.29	0.199	2.143	-0.25	0.802
SGLT2i	1.338	1.03	0.302	1.276	0.68	0.498	1.255	0.86	0.392	1.340	0.18	0.858
α-GI	1.174	0.84	0.401	1.164	1.08	0.282	1.163	0.69	0.491	1.172	1.33	0.185

Pioglitazone	1.087	-1.87	0.062	1.079	1.04	0.299	1.080	0.49	0.625	1.092	2.00	0.046
Insulin	1.380	-0.92	0.358	1.121	-1.92	0.056	1.119	-1.08	0.282	1.397	-1.71	0.088
GLP-1RA	1.547	2.29	0.022	1.444	-2.06	0.040	1.446	0.54	0.590	1.555	-1.63	0.103
